# Whole Genome Sequencing and Molecular Epidemiology of Clinical Isolates of *Staphylococcus aureus* from Algeria

**DOI:** 10.3390/microorganisms11082047

**Published:** 2023-08-09

**Authors:** Rachida Namoune, Abla Djebbar, Rebecca Mekler, Martin McHugh, Mohammed El Amine Bekara, Arun Decano, Matthew T. G. Holden, Mohammed Sebaihia

**Affiliations:** 1 Laboratory of Molecular Biology, Genomics and Bioinformatics, Department of Biology, Faculty of Nature and Life Sciences, University Hassiba Benbouali of Chlef, Chlef 02000, Algeria; r.namoune@univ-chlef.dz (R.N.); ab.djebbar@univ-chlef.dz (A.D.);; 2 School of Medicine, University of St Andrews, St Andrews KY16 9TF, UKmtgh@st-andrews.ac.uk (M.T.G.H.)

**Keywords:** *Staphylococcus aureus*, MRSA, WGS, MLST, antibiotic resistance, Algeria

## Abstract

*Staphylococcus aureus* is an important pathogen responsible for various healthcare- and community-acquired infections. In this study, whole genome sequencing (WGS) was used to genotype *S. aureus* clinical isolates from two hospitals in Algeria and to characterize their genetic determinants of antimicrobial resistance. Seventeen *S. aureus* isolates were included in this study. WGS, single-nucleotide polymorphism (SNP)-based phylogenetic analysis, in silico multilocus sequence typing (MLST), *spa* and staphylococcal cassette chromosome *mec* (SCC*mec*) typing and in silico antimicrobial resistance profiling were performed. Phenotypic antibiotic susceptibility testing was performed using the Vitek 2 system and the disk diffusion method. The isolates were separated into sequence types (STs), with ST80 being predominant; five clonal complexes (CCs); four *spa* types (t044, t127, t368, t386); and two SCC*mec* types (IVc and IVa). Whole genome analysis revealed the presence of the resistance genes *mecA*, *blaZ*, *ermC*, *fusB*, *fusC*, *tetK*, *aph*(3′)-IIIa and *aad*(6) and mutations conferring resistance in the genes *parC* and *fusA*. The rate of multidrug resistance (MDR) was 64%. This work provides a high-resolution characterization of methicillin-resistant *S. aureus* (MRSA) and methicillin-susceptible *S. aureus* (MSSA) isolates and emphasizes the importance of continuous surveillance to monitor the spread of *S. aureus* in healthcare settings in the country.

## 1. Introduction

*S. aureus* is the leading cause of nosocomial infections worldwide [1]. Although it is a normal resident of the skin and mucous membranes of humans and animals, *S. aureus* can become an opportunistic pathogen by deploying a plethora of virulence factors to cause a variety of infections, ranging from mild skin and soft tissue infections to severe and life-threatening diseases, such as toxic shock syndrome and sepsis [2]. This strong potential of *S. aureus* to cause diseases is aggravated by its propensity to acquire resistance to multiple antibiotics, limiting the therapeutic options against this pathogen [3]. 

MRSA is a group of *S. aureus* strains that have developed resistance to methicillin and to the majority of the β-lactam antibiotics following the acquisition of a *mecA* gene. This gene which resides on a complex mobile genetic element known as “Staphylococcal Cassette Chromosome” *mec* element (SCC*mec*) encodes a 78 kDa penicillin-binding protein (PBP2a) that has a low affinity for β-lactams [3]. MRSA is recognized as one of the leading pathogens responsible for nosocomial and community-associated infections worldwide [4]. Effective MRSA control and prevention strategies in the healthcare system as well as in the community depend on accurate characterization of circulating MRSA clones and identification of their reservoirs and sources of transmission. 

Although the prevalence and molecular epidemiology of MRSA in Europe and North America have been extensively documented [5], comparatively, data available from North Africa are limited or scarce. However, from the available data, it appears that the prevalence of MRSA in this region is increasing [6,7]. 

In Algeria, analyses of *S. aureus* from diverse geographic locations and clinical origins were mainly based on genotyping methods. These studies showed that the dominant clone was the European ST80 IV PVL+ community-associated (CA)-MRSA [8,9,10,11]; however, WGS-based studies were applied only in a single study on three healthcare-associated (HA)-MRSA isolates [11]. Thus, here we report the characterization of MRSA and MSSA clinical isolates from patients admitted to two tertiary healthcare hospitals in the province of Chlef in Algeria using WGS.

## 2. Materials and Methods

### 2.1. Study Setting and Sample Collection

We investigated 17 clinical *S. aureus* isolates from pus, cerebrospinal fluid (CSF) and blood samples of patients admitted to two hospitals in the Chlef province, Algeria, during the period between November 2018 and July 2019. 

All patients gave their informed consent for inclusion before they participated in the study. The study was conducted in accordance with the Declaration of Helsinki, and the protocol was approved by the Ethics Committee of the Algerian Thematic Research Agency for Health and Life Sciences (ATRSSV387).

### 2.2. S. aureus Culture and Identification

Bacteria were enriched in brain heart infusion broth and incubated at 37 °C for 18 h–24 h before being cultured on Mannitol salt agar (Merck, Darmastadt, Germany) for 24 h at 37 °C. Isolates were initially identified by standard microbiological methods using Gram staining, catalase and coagulase tests. Putative staphylococcal isolates were confirmed as *S. aureus* by PCR targeting the *nuc* gene as described by Zhang et al. [12]. The PCR reaction was carried out in a total volume of 25 μL, containing 5 μL of chromosomal DNA, 3 μL of each primer (10 μM), 0.5 μL of mixed dNTPs, 2.5 μL of buffer (10×), 0.25 μL of Taq polymerase and 10.75 μL of ultra-pure H_2_O. The PCR amplification program was as follows: an initial denaturation at 94 °C for 10 min, followed by 35 cycles of 94 °C for 1 min, 58 °C for 30 s and 72 °C for 1 min and a final extension step at 72 °C for 7 min. The PCR products were separated on 2% agarose stained with ethidium bromide and visualized using a UV transilluminator. 

### 2.3. Genomic DNA Extraction and Whole Genome Sequencing

The *S. aureus* isolates were grown overnight at 37 °C on tryptic soy broth medium under agitation. Whole DNA extraction was performed using MasterPure Gram Positive DNA Purification Kit (Lucigen, Middleton, WI, USA). The quality of the DNA was assessed with a Qubit^®^ fluorometer 3.0 (Invitrogen, Life Technologies, Singapore). Genomic DNA libraries were prepared using the Nextera XT Library Prep Kit (Illumina, San Diego, CA, USA) and were sequenced on the Illumina MiSeq platform (Illumina, San Diego, CA, USA). De novo assembly was performed using the normal mode of Unicycler pipeline v.0.4.7 [13]. The quality of the assembly was assessed by using Quast 4.6, and the assembly was then annotated with the Prokka software version 1.10 [14], using the default parameters. 

### 2.4. MLST Analysis

In silico MLST of the isolates was performed with the Short Read Sequence Typing for bacterial pathogens (SRST2) program [15], which employs bowtie2 to call loci directly from Illumina reads based on the *S*. *aureus* MLST database PubMLST (https://pubmlst.org/saureus/, accessed on 15 November 2022). 

### 2.5. SNP-Based Phylogenetic Analysis

The assembled complete genomes were aligned to a reference genome of the *S. aureus* strain MSSA476 (an MSSA isolate belonging to ST1, with accession number BX571858 retrieved from EMBL/GenBank databases) using snp-sites (https://github.com/sanger-pathogens/snp-sites, accessed on 15 November 2022) [16]. Pangenome analysis was performed using the Roary pipeline (version 3.11.2) [17], and SNPs were identified using Gubbins software v2.3.2 [18]. A maximal likelihood tree was constructed using RAxML version 8.0.26 [19].

### 2.6. SCCmec and Spa Typing

The SCC*mec* chromosomal cassettes were identified in the genomes using SCC*mec*Finder v.1.2 [20], with minimum thresholds set at 60% for sequence coverage and at 90% for sequence identity. Spa typing was performed using spaTyper v.1.0 [21], using the default parameters.

### 2.7. Analysis of Virulence-Associated Genes 

The Panton–Valentine leukocidin (PVL) toxin (*lukF-PV* and *lukS-PV*) and the toxic shock syndrome toxin 1 (TSST-1) genes were identified by querying the genome sequences against the database Pathosystems Resource Integration Center (PATRIC version 3.6.12: https://www.patricbrc.org/, accessed on 10 December 2022) with the VFDB and Victors tools [22].

### 2.8. Genotypic Antimicrobial Resistance Analysis

The presence of antimicrobial resistance genes (ARGs) was assessed by screening the whole genomes against the Comprehensive Antibiotic Resistance Database (CARD) [23].

### 2.9. In Vitro Antimicrobial Susceptibility Testing 

Microdilution susceptibility testing of isolates was performed using either the Vitek^®^ 2 system (bioMérieux^®^, Marcy L’Etoile, France) or the disk diffusion method. The Vitek^®^ 2 was used with the following antibiotics: erythromycin (ERY), clindamycin (CLI), levofloxacin (LVX), linezolid (LZD), moxifloxacin (MXF), nitrofurantoin (NIT), quinupristin/dalfopristin (QD), tigecycline (TGC), teicoplanin (TEC), tetracycline (TET), trimethroprime + sulfamethoxazol (SXT) and vancomycin (VAN). 

The disk diffusion method on Mueller–Hinton agar was performed with the following antibiotic disks: amikacin (AMK; 30 μg), cefoxitin (FOX; 30 μg), ciprofloxacin (CIP; 5 μg), chloramphenicol (CHL; 30 μg), fusidic acid (FUS; 10 μg), gentamycin (GEN; 10 μg), kanamycin (KAN; 30 μg), ofloxacin (OFX; 5 μg) and rifamycin (RIF; 5 μg). Results were interpreted according to the Clinical and Laboratory Standards Institute guidelines [24] and the Committee of the Antibiogram of the French Society of Microbiology [25]. MDR was defined as resistance to three or more classes of antibiotics.

## 3. Results

### 3.1. Identification of S. aureus

This study included 17 clinical isolates of *S. aureus*, isolated from pus (*n* = 15, 88%), CSF and blood (*n* = 1, 6%, each). The demographic and clinical data of patients as well as the characteristics of the *S. aureus* isolates used in this study are presented in Table 1. The majority of the isolates (58.8%, *n* = 10) were from patients admitted for more than 48 h and were therefore considered as HA-*S. aureus*, according to the CDC definition [26]. Twelve (70.6%) of the *S. aureus* isolates were from adults and five were from children (29.4%); of these isolates, eight (47.1%) were from males and nine (52.9%) were from females. Out of the 17 *S. aureus* isolates, 7 (41.2%) were identified as MRSA by being resistant to cefoxitin and harboring the *mecA* gene, and 10 (85.8%) were MSSA (Table 1).

### 3.2. MLST Analysis

**Figure 1:**
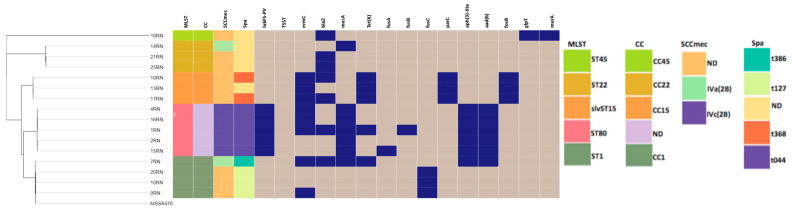
Core genome maximum likelihood phylogenetic tree of the 17 *S. aureus* isolates and the reference genome of the *S. aureus* strain MSSA476, visualized using Phandango [27]. The phylogeny tree on the left is linked to the metadata in the middle: STs, CCs, SCC*mec* types, Spa types (represented by various colors as indicated in the legend on the right); presence (dark blue) and absence (grey) of PVL, TSST and antibiotic resistance gene/mutation.

In silico MLST analysis of the *S. aureus* isolates revealed four different STs, ST1, ST22, ST45 and ST80, and a novel single-locus variant of ST15 (slvST15). ST80 was the most prevalent (*n* = 5, 29.4%), followed by ST1 and ST22 (*n* = 4, 23.5%, each), slvST15 (*n* = 3, 17.6%) and ST45 (*n* = 1, 5.8%) (Figure 1). The MRSA isolates were distributed across three STs, the majority (71.4%, 5/7) in ST80 and one each (14.3%) in ST1 and ST22. The isolates belonged to five known CCs, CC1 (ST1), CC22 (ST22), CC45 (ST45), CC15 (slvST15) and CC80 (ST80) (Figure 1). 

### 3.3. SCCmec, Spa and PVL Typing

A total of four *spa* types, t044 (*n* = 5, 29.4%), t127 (*n* = 3, 17.6%), t368 (*n* = 2, 11.7%) and t386 (*n* = 1, 5.9%), were detected among 11 *S. aureus* isolates; the *spa* type of the six remaining *S. aureus* isolates could not be determined (Figure 1). Among the MRSA isolates, two types of SCC*mec*, type IVc (*n* = 5, 71.4%) and IVa (*n* = 2, 28.6%), have been identified (Figure 1). The genes encoding the two components of the PVL toxin (*lukF-PV* and *lukS-PV*) were detected in five (1RN, 2RN, 4RN, 15RN and 16RN) out of the seven MRSA isolates, all of which belong to ST80 (71%). Notably, the gene encoding the TSST-1 was detected in one isolate (29RN) belonging to ST22. 

### 3.4. Phylogenetic Analysis

Core genome SNP-based phylogeny revealed that the SNP differences among the 17 *S. aureus* isolates ranged from 0 to 60210. Phylogenetic reconstruction grouped the isolates into five separate clusters which matched their respective STs (Figure 1).

### 3.5. Phenotypic Antimicrobial Susceptibility Testing

The 17 isolates were tested for susceptibility against a panel of 21 antibiotics belonging to 13 classes. All *S. aureus* isolates were susceptible to VAN, QD, SXT, LZD, TEC, TGC and NIT. 

The resistance rates observed in this study included 53% to ERY, CLI, AMK and KAN (*n* = 9, each); 47% to FUS (*n* = 8); 41.2% to FOX (*n* = 7); 35% to OFX and TET (*n* = 6 each); 23.5% to CIP (*n* = 4); 17.6% to GEN (*n* = 3); 11.7% to RIF (*n* = 2); and 5.9% to CHL (*n* = 1) (Table 2).

Three isolates exhibited co-resistance to two antibiotic classes (β-lactams and aminoglycosides or β-lactams and fusidic acid), whereas three isolates were resistant to β-lactam antibiotics only.

More than half of the *S. aureus* isolates (64.7%, *n* = 11) displayed multidrug resistance (MDR), displaying resistance to three or more antibiotic classes. The rate of MDR in the MRSA isolates (71.42%, 5/7) was higher than that in the MSSA isolates (60%, 6/10) (Table 2).

### 3.6. In Silico Analysis of Antimicrobial Resistance Genes 

Analysis of the genomes of the 17 *S. aureus* isolates revealed the presence and distribution of 10 acquired antibiotic resistance genes conferring resistance to β-lactams (*blaZ* and *mecA*), erythromycin (*ermC*), tetracycline (*tet(K)*), fosfomycin (*fosB*), fusidic acid (*fusB* and *fusC*), aminoglycosides (*aad(6)* and *aph(3′)-IIIa*) and streptothrin (*sat-4*) (Figure 1) and mutations in four core genes conferring resistance to fusidic acid (*fusA*), fosfomycin (*glpT* and *murA*) and fluoroquinolones *(parC)*. 

Seven *S. aureus* isolates (41%) carried the methicillin resistance gene *mecA* and were therefore considered as MRSA. 

The *ermC* gene encoding a 23S rRNA adenine-N-6 methyltransferase that confers resistance to the macrolide–lincosamide–streptogramin B (MLSB) class of antibiotics was the most frequent antibiotic resistance genetic determinant identified in our collection of isolates, being present in eight isolates (47%). The *blaZ* gene encoding a type C β-lactamase was present in seven (41%) *S. aureus* isolates. The *fusC* or *fusB* genes encoding proteins that block the binding of fusidic acid to EF-G responsible for fusidic acid resistance were identified in four isolates (23.5%), whereas fusidic acid resistance conferred by a mutation H457Q in *fusA* was detected in one isolate (5.8%), 15RN (ST80) (Figure 1).

The fosfomycine resistance gene *fosB*, encoding a fosfomycin-modifying enzyme, was detected in three isolates (17.6%), and a fourth isolate, 19RN (ST45), had mutations A100V in *glpT* and E291D and T396N in *murA*.

Three isolates (17.6%) harbored an S80F mutation in *parC*, conferring resistance to fluoroquinolones (Figure 1).

Of note, the majority of the above resistance genetic determinants (*mecA*, *blaZ*, *ermC*, *fusB*, *tetK*, *aad(6)*, *aph(3′)-IIIa*) were carried by one HA-MRSA isolate (1RN), which had MDR and carried the PVL toxin genes *lukF*/*lukS*-PV.

### 3.7. Correlation between Phenotypic Antimicrobial Susceptibility Testing and Genotype

The observed correlation between phenotypic resistance and genotype varied widely. It was high for FOX/*mecA* (100%), ERY/CLI/*ermC* (94%), TET/*tet(K)* (94%), MOX/*parC* (82%) and LVX/*parC* (76%); moderate for AMK/GEN/KAN/*aph(3′)-IIIa*/*aad(6)* (64-70%); and poor for FUS/*fusB*/*fusC* and OFX/*parC* (58%), CIP/*parC* (52%), QD/*ermC* and FUS/*fusA* (47%).

## 4. Discussion

In this study, WGS was used to determine the phylogeny and molecular characteristics of 10 MSSA and 7 MRSA isolated from clinical samples. The percentage of MRSA among *S. aureus* isolates in our study (41.1%, 7/17) was in line with that reported in North African countries, 31% in Libya, 45% in Algeria and Tunisia and 52% in Egypt [7]. The prevalence of MRSA in Europe varied from 2% in the Netherlands to 58% in Italy [28,29], and that in Asia varied from 22.6% (India) to 86.5% (Sri Lanka) [28], with an average of 25% in both continents [28,30].

The results presented in this study revealed diversity among our collection of *S. aureus* isolates with the isolates falling to one of five clonal complexes. The MRSA clone ST80-*spa*-t044-SCC*mec*-IVc(2b)-PVL+ which was predominant in this study was also reported as dominant in many parts of the world as a cause of infections in both hospital and community settings [31]. This clone was also previously identified as dominant in different ecological niches in Algeria [32,33].

The second most abundant clone, ST1-t127, was identified in the 1990s as the first CA-MRSA clone [34] and then emerged in diverse healthcare settings [35,36,37], and it was also recovered from companion animals, livestock and livestock products in several countries [38,39,40]. Interestingly, the ST1-t127 clone, which was mainly found as MRSA in many studies [41], was both MRSA and MSSA in this study.

The other second most predominant clone in our study, ST22, was represented by three MSSA and one MRSA (SCC*mec* type IVa). MRSA belonging to ST22 (EMRSA-15) were reported as the most frequently responsible for nosocomial infections in Europe, are becoming widespread in Asia, Australia, Europe and North America [42,43,44,45], and have also been sporadically isolated in Algeria, Tunisia and South Africa [46]. However, the ST22 MRSA identified in this study does not belong to the EMRSA-15 clone as it contains a type IVa SCC*mec* element rather than a type IVh and is also ciprofloxacin-sensitive, whereas EMRSA-15 is ciprofloxacin-resistant [42].

Our least frequent clone, ST45, which was commonly reported in North America, Australia and Europe as both MSSA and MRSA in both healthcare and community settings, was also less frequently isolated in South America, Asia and Africa [47].

The SNP-based analysis revealed that the ST1 isolates (10RN and 20RN) were indistinguishable (0 SNP differences). Interestingly, these isolates were recovered from two patients who were admitted 19 days apart to the same hospital and ward (internal medicine), suggesting ward contamination and intra-ward transmission between patients.

Similarly, all the isolates of the ST80 lineage were isolated from the same hospital, two of which (1RN and 16RN) differed by six SNPs only and were from two different patients admitted to the same ward (trauma), but 30 days apart. Isolates 2RN and 15RN, also having a six-SNP difference, were, isolated from patients admitted 45 days apart to separate wards in the same hospital. Isolates 2RN and 4RN, which differed by five SNPs, were isolated from two children admitted 15 days apart to the same ward. The identification of these genomic clusters potentially suggests a persistent contamination of the hospital by the above three clones.

On the other hand, the ST22 isolates (21RN and 25RN) differed by two SNPs; however, they were recovered 51 days apart from two different patients admitted to different hospitals. Considering that (i) the two hospitals are in close proximity (within the same catchment area), (ii) a patient’s choice of being re-admitted to a particular hospital is not restricted, (iii) patients’ health records are decentralized and (iv) inter-hospital patient transfers (for diagnostic procedure or for extra medical care) are frequent, it is therefore possible that any of the above types of patients’ movements and/or referrals between hospitals may have contributed to the dissemination of the *S. aureus* clones 21RN and 25RN between the two hospitals. Indeed, patient movements between healthcare facilities have been recognized as an important route of pathogen transmission [48].

The three CC15 isolates 13RN, 17RN and 18RN were also closely related, with 7-SNP differences between 13RN and 17RN and 17RN and 18RN and an 11-SNP difference between 13RN and 18RN. These three isolates were recovered from different patients admitted to different wards in the same hospital, suggesting an inter-ward transmission in the same hospital.

Irrespective of the exact mode of intra- or inter-hospital transmissions highlighted above, all the possible scenarios should be considered as breaches in infection control and prevention.

The antibiotic sensitivity testing revealed that all the *S. aureus* isolates exhibited susceptibility to VAN, QD (a valuable alternative to vancomycin for the treatment of MRSA infections), SXT, LZD, TEC, TGC and NIT. A similar antibiotic susceptibility profile was also reported in a Kenyan study [49].

The prevalence of vancomycin resistance varied globally from 1% in Europe, 3% in South America and 4% in North America to 5% in Asia and 16% in Africa. Similarly, the susceptibility of all our isolates to SXT was interesting, as a high resistance level to this antibiotic was reported among MRSA isolates in Latin America (up to 100%), Taiwan (89%), China (21%) and Africa (from 55% to 72%) [50].

Overall, the rate of MDR in the present study was relatively high (64%), which is consistent with previous reports from Algeria [11,51,52].

The global prevalence of PVL among MRSA strains varied remarkably between geographical regions and populations [53]. The prevalence of PVL among our MRSA isolates (29.4%) falls within the range of other studies [54]. While PVL is generally considered as a potential genetic marker for the differentiation of CA-MRSA and HA-MRSA [55,56], it was, however, equally present in CA-MRSA and HA-MRSA isolates in the present investigation, which was also consistent with a study from Uganda [57].

## 5. Conclusions

This study suffers from some limitations, mainly the small sample size; despite this, it provides preliminary insights into the genetic diversity and antibiotic resistance of *S. aureus* strains circulating in hospital settings in Algeria. To the best of our knowledge, this is the first WGS-based study that included a relatively large collection of clinical *S. aureus* isolates from Algeria, a country where surveillance of *S. aureus* has been limited thus far. Our findings stress the need for effective MRSA control and prevention strategies in the Algerian healthcare system. In addition, this work highlights the importance of WGS as a useful approach in clinical settings, as it provides high-resolution analyses of pathogens, allowing the determination of the relatedness between epidemic strains and the tracing of their transmission events.

## Figures and Tables

**Table 1:** Patient and *S. aureus* isolate characteristics.

Isolate	MSSA/MRSA	HA/CA	Age	Sex	Admission Date	Collection Date	Hospital	Ward	Specimen
1RN	MRSA	HA	AD	F	20.12.2018	24.12.2018	A	Trauma	Pus
2RN	MRSA	HA	CH	F	15.01.2019	20.01.2019	A	Pediatric	Pus
4RN	MRSA	CA	CH	M	30.12.2018	31.12.2018	A	Pediatric	Pus
7RN	MRSA	HA	AD	F	17.01.2019	20.01.2019	A	Emergency	Pus
9RN	MSSA	CA	CH	M	26.02.2019	27.02.2019	A	Pediatric	Blood
10RN	MSSA	CA	AD	M	21.04.2019	22.04.2019	A	IM	Pus
13RN	MSSA	HA	AD	F	18.04.2019	21.04.2019	A	IM	CSF
14RN	MRSA	CA	CH	M	22.01.2019	23.01.2019	A	Pediatric	Pus
15RN	MRSA	CA	AD	F	28.11.2018	29.11.2018	A	Emergency	Pus
16RN	MRSA	CA	AD	M	21.01.2019	22.01.2019	A	Trauma	Pus
17RN	MSSA	HA	CH	F	11.02.2019	24.02.2019	A	Pediatric	Pus
18RN	MSSA	HA	AD	F	14.02.2019	25.02.2019	A	Emergency	Pus
19RN	MSSA	CA	AD	M	13.03.2019	14.03.2019	B	ENT	Pus
20RN	MSSA	HA	AD	M	09.05.2019	13.05.2019	A	IM	Pus
21RN	MSSA	HA	AD	F	09.05.2019	13.05.2019	A	IM	Pus
25RN	MSSA	HA	AD	F	21.03.2019	24.03.2019	B	IM	Pus
29RN	MSSA	HA	AD	M	23.04.2019	29.04.2019	A	IM	Pus

MRSA: Methicillin-Resistant *S. aureus;* MSSA: Methicillin-Susceptible *S. aureus*; CA: Community-Associated; HA: Healthcare-Associated; AD: Adult; CH: Child; F: Female; M: Male; IM: Internal Medicine; ENT: Ear, Nose and Throat; CSF: Cerebrospinal Fluid.

**Table 2:** Phenotypic antimicrobial resistance profiles of the *S. aureus* isolates.

Isolate	MIC (μg/mL)												Inhibition Zone Diameters (mm)									
	LVX	MXF	ERY	CLIN	QD	LZD	TEC	VAN	TET	TGC	NIT	SXT	CHL	GEN	AMK	KAN	FOX	CIP	OFX	FUS	RIF	MDR
1RN	S	S	R	R	S	S	S	S	R	S	S	S	S	S	R	R	R	I	R	R	S	Y
2RN	S	S	S	S	S	S	S	S	S	S	S	S	S	S	R	R	R	R	S	R	S	Y
4RN	S	S	R	R	S	S	S	S	S	S	S	S	S	R	R	R	R	I	R	S	R	Y
7RN	S	S	R	R	S	S	S	S	R	S	S	S	S	S	S	R	R	S	S	S	S	Y
9RN	S	S	R	R	S	S	S	S	S	S	S	S	S	S	S	S	S	S	S	R	S	Y
10RN	S	S	S	S	S	S	S	S	S	S	S	S	S	S	S	S	S	I	S	S	I	N
13RN	S	S	R	R	S	S	S	S	R	S	S	S	S	R	R	R	S	R	S	S	I	Y
14RN	S	S	S	S	S	S	S	S	S	S	S	S	S	S	I	I	R	S	S	S	S	N
15RN	S	S	S	S	S	S	S	S	S	S	S	S	S	S	R	R	R	S	S	S	S	N
16RN	S	S	R	R	S	S	S	S	R	S	S	S	S	R	R	I	R	I	R	R	S	Y
17RN	S	S	R	R	S	S	S	S	R	S	S	S	S	S	R	R	S	R	R	S	S	Y
18RN	S	S	R	R	S	S	S	S	R	S	S	S	R	S	R	R	S	I	S	R	S	Y
19RN	S	S	R	R	S	S	S	S	S	S	I	S	S	S	S	S	S	I	R	S	R	Y
20RN	S	S	S	S	S	S	S	S	S	S	S	S	S	S	R	R	S	R	R	R	S	Y
21RN	S	S	S	S	S	S	S	S	S	S	S	S	S	S	S	S	S	S	S	S	S	N
25RN	S	S	S	S	S	S	S	S	S	S	S	S	S	S	S	S	S	S	S	R	S	N
29RN	I	S	S	S	S	S	S	S	S	S	S	S	S	S	S	S	S	S	S	R	S	N
Total																						
*n*	0	0	9	9	0	0	0	0	6	0	0	0	1	3	9	9	7	4	6	8	2	11
(%)	(0%)	(0%)	(53%)	(53%)	(0%)	(0%)	(0%)	(0%)	(35%)	(0%)	(0%)	(0%)	(5.9%)	(17.6%)	(53%)	(53%)	(41.2%)	(23.5%)	(35%)	(47%)	(11.7%)	(64.7%)

MIC: Minimum Inhibitory Concentration (μg/mL); *n*: Number; R: Resistant; I: Intermediate; S: Susceptible; MDR: Multidrug Resistance; Y: Yes; N: No; AMK: Amikacin; CHL: Chloramphenicol; CIP: Ciprofloxacin; CLIN: Clindamycin; ERY: Erythromycin; FOX: Cefoxitin; FUS: Fusidic Acid; GEN: Gentamycin; KAN: Kanamycin; LVX: Levofloxacin; LZD: Linezolid; MXF: Moxifloxacin; NIT: Nitrofrurantoin; OFX: Ofloxacin; QD: Quinupristin/Dalfopristin; RIF: Rifamycin; SXT: Trimethroprim/Sulfamethoxazole; TEC: Teicoplanin; TET: Tetracycline; TGC: Tigecyclin; VAN: Vancomycin.

## Data Availability

Raw reads from Illumina sequencing generated in this study were deposited in the NCBI Sequence Read Archive (SRA) under the BioProject accession number PRJNA930663.
